# Novel Structurally Designed Vaccine for *S. aureus* α-Hemolysin: Protection against Bacteremia and Pneumonia

**DOI:** 10.1371/journal.pone.0038567

**Published:** 2012-06-06

**Authors:** Rajan P. Adhikari, Hatice Karauzum, Jawad Sarwar, Laura Abaandou, Mahta Mahmoudieh, Atefeh R. Boroun, Hong Vu, Tam Nguyen, V. Sathya Devi, Sergey Shulenin, Kelly L. Warfield, M. Javad Aman

**Affiliations:** Integrated Biotherapeutics Inc., Gaithersburg, Maryland, United States of America; National Institutes of Health, United States of America

## Abstract

*Staphylococcus aureus* (*S*. *aureus*) is a human pathogen associated with skin and soft tissue infections (SSTI) and life threatening sepsis and pneumonia. Efforts to develop effective vaccines against *S. aureus* have been largely unsuccessful, in part due to the variety of virulence factors produced by this organism. *S. aureus* alpha-hemolysin (Hla) is a pore-forming toxin expressed by most *S. aureus* strains and reported to play a key role in the pathogenesis of SSTI and pneumonia. Here we report a novel recombinant subunit vaccine candidate for Hla, rationally designed based on the heptameric crystal structure. This vaccine candidate, denoted AT-62aa, was tested in pneumonia and bacteremia infection models using *S. aureus* strain Newman and the pandemic strain USA300 (LAC). Significant protection from lethal bacteremia/sepsis and pneumonia was observed upon vaccination with AT-62aa along with a Glucopyranosyl Lipid Adjuvant-Stable Emulsion (GLA-SE) that is currently in clinical trials. Passive transfer of rabbit immunoglobulin against AT-62aa (AT62-IgG) protected mice against intraperitoneal and intranasal challenge with USA300 and produced significant reduction in bacterial burden in blood, spleen, kidney, and lungs. Our Hla-based vaccine is the first to be reported to reduce bacterial dissemination and to provide protection in a sepsis model of *S. aureus* infection. AT62-IgG and sera from vaccinated mice effectively neutralized the toxin *in vitro* and AT62-IgG inhibited the formation of Hla heptamers, suggesting antibody-mediated neutralization as the primary mechanism of action. This remarkable efficacy makes this Hla-based vaccine a prime candidate for inclusion in future multivalent *S. aureus* vaccine. Furthermore, identification of protective epitopes within AT-62aa could lead to novel immunotherapy for *S. aureus* infection.

## Introduction

*Staphylococcus aureus* (*S. aureus*) is a ubiquitous, formidable Gram-positive pathogen associated with skin and soft tissue infections (SSTI), as well as life threatening sepsis and pneumonia [1]. Since its first emergence in the 1960s methicillin-resistant *S. aureus* (MRSA) has become endemic in hospitals and healthcare settings worldwide [2]. Since the 1990s, several community associated MRSA strains (CA-MRSA) have emerged and are spreading worldwide, posing a major global challenge [3], [4], [5]. There are currently no vaccines available for the prevention of S. aureus infections. The pathogenicity of S. aureus is dependent on numerous virulence factors, including cell surface proteins, polysaccharides, and secreted toxins. The latter cause tissue damage, promote bacterial dissemination and metastatic growth in distant organs, and allow the pathogen to evade the host innate immune response [6], [7]. The pore-forming α-hemolysin (Hla), also known as α-toxin (AT), is produced by nearly all virulent strains and is implicated in several *S. aureus* diseases including SSTI [8] and pneumonia [9].

Several lines of evidence validate Hla as an important vaccine target for prevention of *S. aureus* infection or complications of disease: i) *hla* is encoded by a chromosomal determinant [10], and its production has been detected in most *S. aureus* isolates [11], [12], [13], [14]; ii) a partially attenuated Hla mutant (H35L) or a truncated Hla protect mice against *S. aureus* pneumonia and skin infections [8], [9],[15]; and iii) passive immunization with antibodies raised against H35L protect mice from lethal toxin challenge and partially protect against bacterial challenge in pneumonia and skin infection models [16]. While the H35 mutation largely abrogates the lytic activity of Hla, a single point mutation is not considered sufficiently safe to be developed as vaccine for human use. Importantly, Panchal *et al* reported that several reverting point mutations can be identified that restore the lytic activity of Hla-H35 mutants [17]. Furthermore, removal of 30 or 99 amino acids at the C terminus of the H35A mutant of Hla reactivated its hemolytic activity [18]. Therefore, there is a need to identify subdomain mutants of Hla with an increased safety profile capable of inducing protective immune responses.

In this study, using a rational, structure-based approach, we designed several truncation mutants of Hla as vaccine candidates and examined their efficacy in two models of *S. aureus* infection. Importantly, this study demonstrates, for the first time, efficacy of a Hla based vaccine candidate against *S. aureus* bacteremia and distant organ bacterial seeding.

## Materials and Methods

### Bacteria

*S. aureus* strain USA300 (Los Angeles County clone, LAC) was obtained from the NARSA repository and *S. aureus* strain Newman was kindly provided by Dr. Tim Foster (Trinity College Dublin, Ireland).

### Preparation of inoculation seeds for pneumonia model

Newman or USA300 strains were grown overnight (ON) in a volume of 20 ml in brain heart infusion (BHI) medium at 37°C, shaking at 230 rpm using a 50 ml culture tube. Multiples of 20 ml cultures were prepared. ON cultures were centrifuged at 3000 rpm and washed twice in PBS using the original volume (20 ml) before pellet was re-suspended in 1 ml phosphate buffered saline (PBS). Multiples of re-suspended pellets were combined and mixed thoroughly on a vortex and further re-suspended with a 28 Gauge needle to keep chain formation of bacterial cells to a minimum. Subsequently 1ml aliquots of seed culture were prepared and stored at −80°C. Three aliquots were streaked out at different dilutions and different time points (to test stability of the seed) to enumerate CFU.

### Preparation of inoculation culture for bacteremia model

For bacterial challenges, CA-MRSA USA300 and USA400 were grown for 18 to 24 hours in Tryptic soy broth (TSB, Difco Laboratories, Detroit, Mich.). 10 ml of TSB in 25 ml flask was inoculated with a single bead of *S. aureus* from −80°C stored bead stock and culture grown ON at 37°C, with shaking at 230 rpm. The culture was centrifuged at 3000 rpm at RT, washed once with PBS and the bacterial pellet re-suspended in 1ml sterile PBS and used for challenges as described below.

### Animals

Female BALB/c mice- 6–8 weeks of age for active immunogenicity studies and 10–12 weeks for passive vaccination studies- were purchased from Charles River laboratories.

Mice were maintained under pathogen-free conditions and fed laboratory chow and water ad libitum. All mouse work was conducted in accordance with protocols approved by institutional animal care and use committees (IACUC) of Nobel Life Sciences (Gaithersburg, MD), where animal studies were conducted.

### Vaccinations

For active immunogenicity studies, mice were immunized intramuscularly (IM) three times at two weeks interval with the antigens formulated in adjuvant. The doses of vaccine and adjuvant are specified for each experiment in the results section. For immunization with Al(OH)_3_ or AlPO_4_ the antigen was pre-absorbed to adjuvant for 1 hour at a ratio of 1∶7 (antigen/adjuvant) in 50 mM Tris, pH 7.5. GLA-SE was mixed with the antigen in PBS before injection. For passive vaccination studies, mice were treated with 4 mg of AT62-IgG in 500 μl volume of PBS via intraperitoneal (IP) administration 24 hours prior to bacterial challenge.

### Mouse pneumonia model

Mice were anesthetized with isoflurane and inoculated intranasally (IN) with a lethal dose of *S. aureus* Newman or USA300 in 50 μl DPBS and were placed into the cages in a prone position until recovery. Animals were monitored for morbidity (weight, hunched posture, labored breathing, ruffled fur, impaired mobility) and mortality 4 times a day within the first 48 hours and then once a day until termination of study.

### Mouse bacteremia model

Female BALB/c mice were challenged via intra-peritoneal (IP) injection with CA-MRSA USA300 in 3% mucin-PBS solution as previously described [19]. Briefly, lypholized hog mucin type III (Sigma Aldrich, St. Louis, Mo) was solublized to 6% (w/v) in PBS, sterilized by autoclaving for 10 minutes and rapidly cooled on ice for 10 – 15 minutes. For bacterial challenges, PBS washed overnight grown USA300 bacterial cells were suspended in PBS to an optical density of 0.15 at 600 nm, corresponding to 7×10^7^ CFU/ml, and then adjusted to 2×10^5^ CFU/ml with PBS. At the time of challenge, bacteria and mucin solution were mixed at equal volumes and mice injected IP with 0.5 ml corresponding to 5×10^4^ CFU in 3% mucin-PBS. Mice were monitored for morbidity and mortality twice a day up for 7 days post challenge.

### Determination of bacterial load during infection

In some experiments, groups of mice were euthanized at 12 h after challenge and blood and organs (liver, combined kidneys, lungs and spleen) were aseptically removed to determine bacterial load. Organs were homogenized with 3.2 mm stainless steel beads using a Bullet Blender from Next Advance Inc. (Averill Park, NY) and were taken up in a total volume of 500 μl PBS. Blood samples and organ homogenates were streaked out in different dilutions on BHI agar plates and CFU were enumerated after ON incubation at 37°C.

### Plasmids, protein expression and purification

Plasmids pET24a (+):35929 (AT79aa-His), pET24a (+):64356 (AT79aa-5G-His), pET24a (+):35930 (AT-62aa-His) and pET24a were synthesized by DNA2.0 (Menlo Park, CA). AT-50aa-His was constructed by cloning the PCR product with *NdeI* and *XhoI* restriction sites into pET24a(+). Forward primer: *ttCATATG*aaaacacgtatagtcagc and reverse primer: ttCTCGAGatcacctgtttttactgtag were used to amplify nucleotide sequences corresponding to the first 50aa of mature alpha toxin protein by using *S. aureus* USA300 DNA template (NARSA, Chantilly, VA). These plasmids were designed to encode the fusion proteins AT79aa with three glycine linker, AT79aa with 5 glycine linker, AT62aa and AT50aa, all possessing a C-terminal 6X Histidine tag. All the alpha toxin constructs were expressed in *Escherichia coli* strain BL21 DE3. For the expression of the protein, bacteria were grown at 37°C and induced with 0.3 mM isopropyl-β-D thiogalactopyranoside (IPTG) when OD_600_ absorption reached 0.5. The temperature was then reduced to 25°C and the induction was continued for another 16 hours. Cells were harvested by centrifugation at 5000 rpm for 20 minutes. The pellet was resuspended in low salt buffer (10 mM Tris-HCl and 20 mM NaCl, pH 7.4) and treated with 0.2 mg/mL Lysozyme. After incubation on ice for 30 minutes, the cells were sonicated in the presence of EDTA free protease inhibitor cocktail (Roche) tablet. After centrifugation at 9000 rpm for 30 minutes, the clarified cell lysate was further treated with 1M NaCl, 2 mM Imidazole and 0.25 % CHAPS and used for protein purification.

The proteins were bound to 5 ml Ni-NTA column (GE Healthcare) on an AKTApurifier® (GE Healthcare) in PBS and washed extensively in the same buffer supplemented with 1 M NaCl and 5 mM imidazole. Elution was performed in 20 column volumes of 0–70% of 1 M imidazole. For AT-79aa, eluted fractions were dialyzed out of imidazole and re-purified one more time on Ni-NTA. AT-50aa and AT-62aa eluted fractions were concentrated with Amicon Ultra 3K filters (Millipore) and purified further by Size Exclusion Chromatography using Superdex 75 10/300 GL (GE Healthcare) column equilibrated in PBS with 0.5 M NaCl and 0.25% CHAPS. The purity and identity of all the eluted alpha toxin variants were confirmed by SDS-PAGE and Western blot using anti-alpha toxin mAb (6C12) generated against AT-79aa construct as the primary antibody and goat anti-mouse as the secondary antibody. The pure proteins were dialyzed in PBS with 10% Glycerol as the storage buffer. The concentrations were determined by bicinchoninic acid (BCA) assay and the endotoxin levels were determined by Limulus Amoebocyte Lysate (LAL) chromogenic endotoxin assay. The proteins were stored at −80°C until further use.

### Molecular modeling

Model building and computer calculations were performed using the Accelrys, Inc software Discovery Studio 2.5 running on a Dell Precision 690 running Red Hat Enterprise Linux 4. Simulations were *in vacuo* and employed a CHARMM force field with a CFF partial charge, a distance-dependent electric constant of 1, and a temperature of 300K. Energy minimization involved 1000 steps of conjugate gradient. The crystal structure of heptameric AT (PDB code 7AHL) [20] was selected for this study. Peptide segments consisting of residues 1–62 and 223–236 were extracted from subunit A of the crystal structure and used as the template for modeling candidate constructs. The peptide segment 1–62 was energy minimized and its molecular energy was calculated as described in Discovery Studio 2.5. Furthermore, the 1–62 peptide segment was used to generate shorter peptide segment, such as 1–50, which were also minimized and used in comparative analysis with one another as candidate constructs based on calculated molecular energies. To generate the 4-strand protein model, the 1–62 and 223–236 segments were bridged with varying number of glycine amino acids between residues 62 and 223, and the resulting structures were subjected to energy minimization and energy calculations. The optimal bridging segment consisting of three glycine amino acids were found to be minimal for forming a stable fold.

### Alpha hemolysin ELISA

Blood samples were centrifuged in serum separator tubes and serum samples were stored at −80°C until further use in ELISA. Briefly, 96-well plates were coated with 100 ng/well of full length alpha toxin (List Biological Laboratories, Campbell, CA) overnight at 4°C. Plates were blocked with Starting Block buffer (Thermo Scientific) for one hour at room temperature (RT). Serum samples were prepared in semi-log dilutions starting from 1∶100 to 1∶10,000.000 in a 96-well plate using starting block buffer as diluent. Plates were washed three times and sample dilutions were applied in 100 μl volume/well. Plates were incubated for one hour at RT and washed three times before applying the conjugate, goat anti-mouse IgG (H&L)-HRP (Horse Radish Peroxidase) in starting block buffer. Plates were incubated for one hour at RT, washed as described above and incubated with TMB (3,3′,5,5′-tetramethylbenzidine) to detect HRP for 30 min. Optical density at 650 nm was measured using a Versamax^TM^ plate reader (Molecular Devices CA). Data analysis for full dilution curves was performed using Softmax program.

### Alpha hemolysin oligomerization inhibition assay

500 µl of 10% rabbit red blood cells (RBC) (Colorado Serum Company, CO) with 15 µg of purified alpha toxin was incubated on ice for 15 min. Following incubation, samples were centrifuged at 14,000 rpm for 5 min in pre-chilled centrifuge. Supernatants were discarded and pellet was washed with cold PBS and then re-suspended in 100 µl of tris-buffered saline (TBS). The samples were incubated at 37°C for 45 min for complete lysis of erythrocytes. Negative (without toxin) and positive (with 1% Triton X-100) controls for hemolysis were run in parallel. The samples were then centrifuged for 10 min at 14,000 rpm and re-suspended in 120 μl of 1× Laemmli buffer, divided into two separate aliquots, one aliquot incubated at 100°C for 10 minutes (negative oligomerization control) and others were kept on ice. 15 μl of each sample was loaded on 7–20% sodium dodecyl sulfate (SDS)-polyacrylamide gels for electrophoresis. For Western blot, rabbit anti-AT-62aa polyclonal antibody (IBT Bioservices Cat#04-0010) was used in a 1∶1000 dilution to detect the monomeric and heptameric bands. For oligomerization inhibition assay, decreasing concentrations of antibody (400–6.25 µg/ml in two-fold dilutions) were incubated with RBC and alpha toxin at RT prior to incubation on ice for 15 min.

### Alpha hemolysin neutralization assay

Alpha hemolysin neutralization titers of mouse sera and rabbit AT-62aa polyclonal antibodies were determined based on neutralization of hemolysis of 2% RBC when pre-incubated with alpha toxin (100 ng) at RT for 10 minutes before adding RBC followed by 30 min incubation at 37°C. After incubation, cells were centrifuged and the absorbance in the supernatant was determined in Versamax^TM^ plate reader (Molecular Devices CA) at 416 nm. Neutralization titer 50 (NT_50_, defined as the dilution of the serum which neutralizes the toxicity of alpha toxin by 50%) was determined by plotting the OD_416nm_ in diluted serum samples using a four parameter logistic (4-PL) curve fit. Standard serum samples with high, medium and low NT_50_ were run to the assay during each assay run.

## Results

### Design of recombinant α-hemolysin vaccine candidates

**Figure 1:**
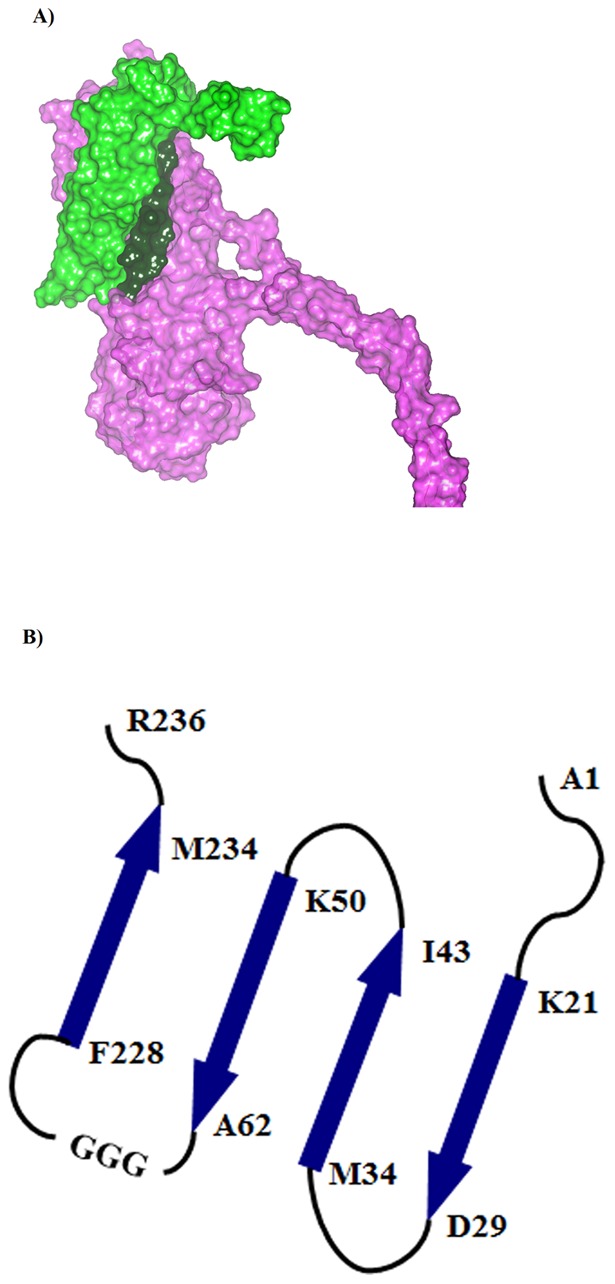
Structural analysis of Hla. (**A**) The relative topology of 1–62 and 1–62(GGG)–(223–236) AT constructs on the protein surface of a subunit from the 7AHL heptameric hemolysin crystal structure. The protein surface for the 1–62 segment is colored green, the 223–236 sequence colored dark green, and the remaining structure colored purple. (**B**) Topology of the secondary structural elements in α-hemolysin for peptide segments examined in this study.

The functional, and cytolytic form of Hla is a pore-forming heptamer that binds to cell surface receptor ADAM10 [21]. Crystallographic studies show that each N-terminus of Hla is located on the top surface of the heptameric pore where it both latches onto a neighboring subunit in an arm-in-arm manner and lines the *cis* (extracellular) entrance to the channel [20]. The N-terminal domain of Hla consists of four anti-parallel β-strands with three of the strands within the linear sequence of 1–62 (residues K21-D29, M34-I43, and K50-A62) and the fourth strand being contributed by the distal amino acids F228-M234 (Figure 1A&B). Given the positioning of the N-terminal domain and previous reports indicating vaccine potential of a truncated N-terminal domain (residues 1–50) [15], we hypothesized that proteins based on stable N-terminal domain structures would produce superior vaccine candidates. Molecular modeling was used to identify optimal fusion proteins based on this four-strand sheet structure. Hypothetical proteins consisting of residues 1–50, 1–62 or a fusion of residues 1–62 to 228–236 (Figure 1B) were examined.

The 1–50, 1–62, and 223–236 segments were extracted from a subunit of the AT crystal structure. The relative stabilities of the 1–50 and 1–62 segments were calculated (Table 1), suggesting that the novel 1–62 construct may be more stable than the reported 1–50 construct [15]. To investigate the potential of the full four-strand sheet structure, molecular modeling was used to evaluate different linker units that could be used to bridge Ala62 in the third strand with Phe228 in the fourth strand, in combination with the loop segment Gly223-Asp227. Because of their small side chain and conformational flexibility, glycine residues were used as the building blocks for the linkers that join Ala62 with Gly223 (Figure 1B). Six different protein models of the 4-stranded sheet structure with varying glycine counts in their linker units were generated and evaluated *in silico*. A three-glycine linker unit had a lower calculated molecular energy than both the 1–50 and 1–62 segments (Table 1). Linkers composed of more than three glycine amino acids are calculated to be less stable than the three-glycine linker construct, but their folding into stable tertiary structure may remain feasible due to the greater conformational flexibility of the larger linkers. The results of this modeling study suggested that a construct consisting of residues 1–62 (denoted hereafter AT-62aa), a construct of residues 1-62-(GGG)-223-236 (denoted hereafter AT-79aa) and constructs containing three or more glycine amino acids would fold into functional domains.

**Table 1:** Calculated Molecular energies for Hla N-terminal constructs.

Construct (residues)	Energy (kcal/mol)
1–50	−2989
1–62	−3660
1–62-(GGG)-223–236	−3953

**Figure 2:**
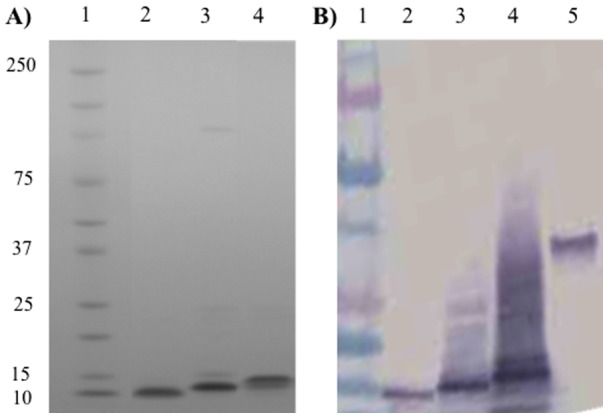
SDS-PAGE and Western blot analysis of purified Hla constructs. (**A**) SDS-PAGE. Lane 1: Biorad pre-stained protein standard; Lane 2: AT-50aa; Lane 3: AT-62aa; Lane 4: AT-79aa. All proteins were loaded at 1 µg/ lane and stained with Coomassie blue. (**B**) Western Blot. Lane 1: Biorad pre-stained protein standard; Lane 2: AT-50aa; Lane 3: AT-62aa; Lane 4: AT-79aa; Lane 5: Full length alpha toxin. All proteins were detected by anti-alpha toxin mAb (6C12) generated against AT-79aa construct.

Based on these predictions, we generated constructs for AT-50aa, AT-62aa, and AT-79aa with a C-terminal 6xHistidine tag. Proteins were expressed in *E. coli* and purified over a Ni column. Based on the molecular modeling study, we prepared constructs consisting of variable number of glycine amino acids, and determined that extension of the linker to five glycines resulted in the best protein expression, yield and purity for *in vivo* studies. Therefore, a five-glycine linker AT-79aa was used in the studies described here. Proteins were analyzed by SDS-PAGE and Western Blot (Figure 2) and tested for endotoxin content as described in the Materials and Methods section.

### Immunogenicity of α-hemolysin vaccine candidates

The immunogenicity of the three purified proteins was examined in BALB/c mice in combination with Alhydrogel (Al(OH)_3_), aluminum phosphate (AlPO_4_), or Glucopyranosyl Lipid Adjuvant-Stable Emulsion (GLA-SE) (ImmuneDesign Corp., Seattle, WA). Groups of five mice were immunized three times at 2 weeks interval with 5 µg of the antigen along with different doses of each adjuvant (Table 2). Mouse sera were tested for total and neutralizing antibodies to wild type Hla on day 35. To evaluate the relationship between immunogenicity and protection from lethal challenge, mice were challenged IP on day 52 with 5×10^4^ CFU of *S. aureus* strain USA300 along with 3% Hog Mucin and monitored for morbidity and mortality over 7 days. Total antibody titers were determined by ELISA using full length purified Hla as coating antigen and seven semi-log dilutions of sera. The ELISA titer (EC_50_) was defined as the dilution of the serum resulting in 50% maximum OD (inflection point of the 4-PL curve). Similarly, the neutralizing titer (NT_50_) was defined as the dilution of the antibody resulting in 50% inhibition of the lysis of rabbit RBCs induced by 100 ng of purified Hla.

**Table 2:** Immunogenicity of AT-62aa tested in BALB/c mice using different adjuvants and survival upon IP challenge with USA300.

Adjuvant	Adjuvant dose	Mouse #	ELISA EC_50_	Neut titer NT_50_	Time of death
		M1	361	<64	20h
		M2	658	<64	survived
Al(OH)_3_	35 μg	M3	198	<64	20h
		M4	69	<64	20h
		M5	75	<64	survived
		*Geo Mean*	*189*	*<64*	
		M1	1230	127	survived
		M2	18	<64	survived
AlPO_4_	35 μg	M3	510	<64	survived
		M4	674	127	survived
		M5	320	110	survived
		*Geo Mean*	*300*		
		M1	1800	251	survived
		M2	1630	194	survived
GLA-SE	20 μg	M3	1530	159	survived
		M4	2540	423	survived
		M5	8170	859	survived
		*Geo Mean*	*2476.5*	*308.9*	
		M1	198	<64.	20h
		M2	208	<64.	20h
No adjuvant	-	M3	91.5	<64.	20h
		M4	76.7	<64.	20h
		M5	307	<64.	20h
		*Geo Mean*	*155*	*<64*	
		M1	0	<64.	20h
Control		M2	0	120	20h
vaccine:	35 μg	M3	0	<64.	20h
(STEBVax)		M4	0	<64.	20h
+ Al(OH)3)		M5	0	<64.	20h

As shown in Table 2, all mice in the no-adjuvant and control groups consistently succumbed within 20 h of USA300 challenge, providing a reliable measure of lethality. Very low ELISA and undetectable NT_50_ titers were observed in mice vaccinated with AT-62aa without adjuvant. Mice vaccinated with a control vaccine (recombinant *Staphylococcal* enterotoxin B vaccine; STEBVax) [22] along with Al(OH)_3_ showed no titer to Hla. In contrast, mice survival was improved by absorption of AT-62aa to Al(OH)_3_. As quantified, Al(OH)_3_ induced low ELISA titers with a geometric mean of 189 and neutralizing titers below the limit of detection. Consistent with the low antibody titers, 3 out of 5 mice in this group succumbed within 20 h of challenge; however, two mice survived and remained active.

Mice survival was markedly improved by using AlPO_4_ or GLA-SE as adjuvants. Mice receiving the vaccine with AlPO_4_ showed slightly higher antibody titers with a geometric mean of 300 and 3 out of 5 mice showed detectable neutralizing titers. All mice in this group survived the challenge and were comparatively less morbid, remaining active with ruffled coat in the first two days post challenge. All mice immunized with AT-62aa along with 20 μg of GLA-SE showed much higher ELISA and NT_50_ titers with geometric means of 2476 and 309 respectively. Consistent with the high titers all mice survived the challenge and remained active. Based on these promising results, we selected GLA-SE as the adjuvant for further comparative efficacy studies.

### Comparative immunogenicity and efficacy of the vaccine candidates

**Figure 3:**
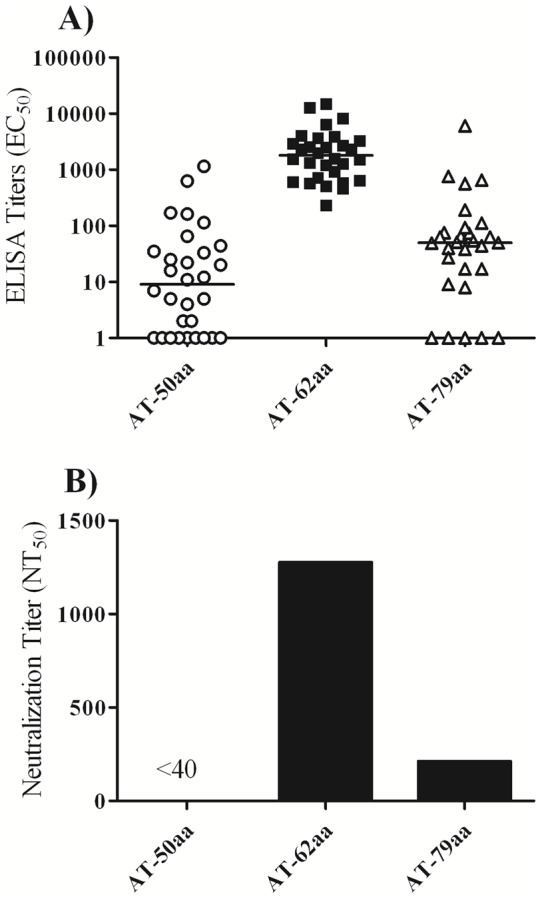
Comparative antibody response to vaccine candidates. (**A**) Individual serum antibody titers towards wild-type Hla determined after three immunizations (**B**) Neutralization titers of pooled sera towards wild-type Hla after three immunizations.

Previous reports using AT-50aa protein indicated the efficacy of this vaccine candidate against pneumonia by the *S. aureus* Newman strain [15], when used with Freund’s adjuvant. Since Freund’s adjuvant cannot be used in humans, we sought to perform a comparative efficacy study with the three vaccine candidates using GLA-SE, an adjuvant currently in clinical development [23], [24]. Groups of 20 mice were vaccinated intramuscularly three times at 2 week intervals with 5 μg of AT-50aa, AT-62aa or AT-79aa, formulated with 5 μg of GLA-SE in PBS (in an interim study we showed that similar titers can be achieved with 5 or 20 μg of GLA-SE; data not shown). On day 35, mice were bled for determination of antibody titers. As shown in Figure 3A, mice vaccinated with AT-62aa showed robust ELISA titers against wild type Hla with median EC_50_ of 2022 (range: 510–14,900). Mice vaccinated with AT-79aa showed much lower ELISA titer with median of 49 (range: 0–6,050) followed by mice vaccinated with AT-50aa with a median EC_50_ of 11 (range: 0–1,150). Similarly, when neutralization titers were determined in pools of serum samples mice vaccinated with AT-62aa showed highest NT_50_ of 1277 followed by AT-79aa with NT_50_ of 213 (`ure 3B). Neutralization was below the limit of detection of this assay in the pool of sera from AT-50aa vaccinated mice (NT_50_<40). For the challenge studies, each group was broken into two subgroups of ten mice each and challenged as described below to determine vaccine efficacy against *S. aureus* pneumonia and sepsis.

#### Efficacy of the vaccine candidates in S. aureus USA300 bacteremia model

**Figure 4:**
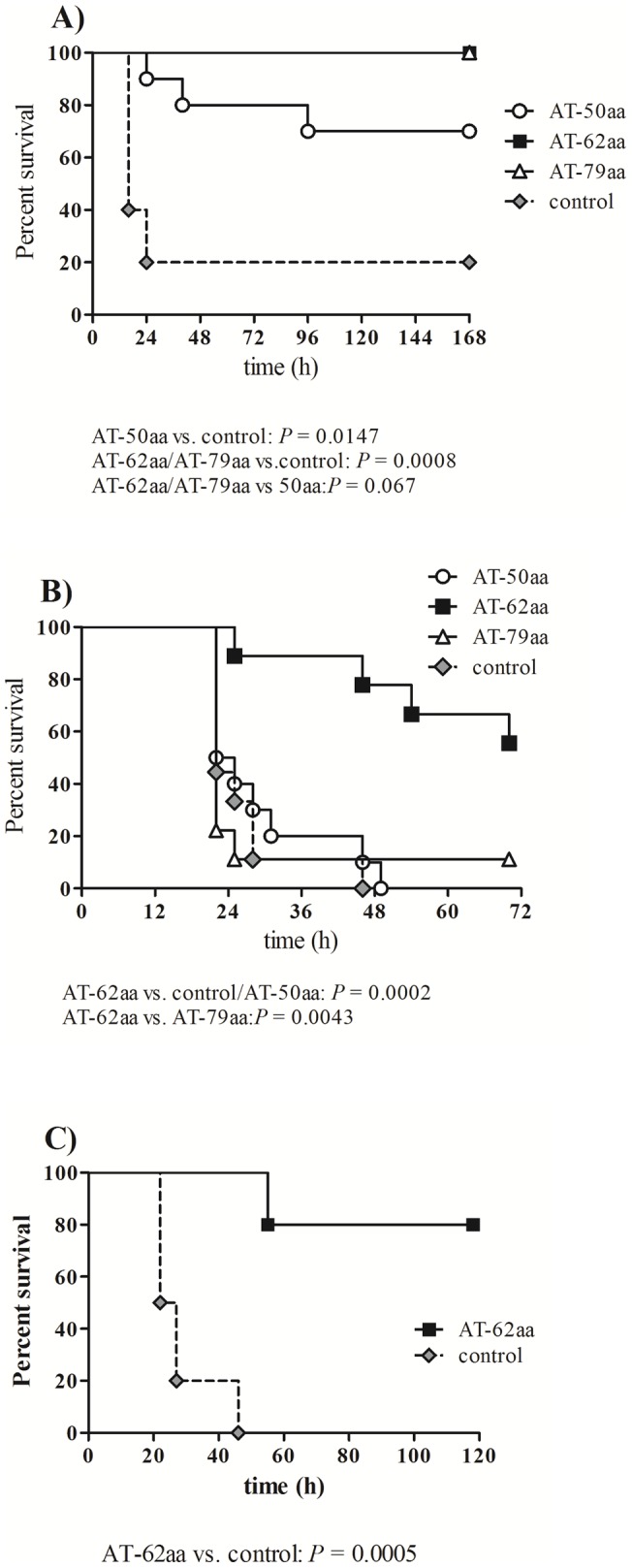
Comparative efficacy study of vaccine candidates in *S. aureus* bacteremia and pneumonia infection models. Survival of mice vaccinated with the three vaccine candidates and control mice after IP challenge with 5×10^4^ CFU of USA300 along with Hog Mucin (**A**) or IN challenge with 6×10^7^ CFU of *S. aureus* strain Newman (**B**). Survival of mice vaccinated with AT-62aa and challenged IN with 1.5×10^8^ CFU of *S. aureus* USA300 (**C**). Symbol key: AT-50aa (open circle), AT-62aa (black square), AT-79aa (open triangles) and mock-immunized mice (grey diamond). Statistical analysis was performed using Log-Rank (Mantel-Cox) test.

Groups of ten vaccinated or five control mice were challenged on day 42 by IP administration of 5×10^4^ CFU of *S. aureus* strain USA300 along with 3% Hog Mucin. Mice were observed for signs of mortality and morbidity for 7 days. While 100% of mice vaccinated with AT-62aa or AT-79aa and 70% of mice vaccinated with AT-50aa survived the challenge, only one out of five control mice survived (Figure 4A). Maximal median weight loss of surviving mice in immunized groups was 6% of body weight. All surviving mice re-gained weight and had a healthy appearance (smooth fur, active) by day 7.

#### Efficacy of the vaccine candidates in S. aureus Newman pneumonia

The remaining ten mice from vaccinated or control groups were challenged on day 48 by intranasal administration of 6×10^7^ CFU of *S. aureus* strain Newman. As shown in Figure 4B, mice vaccinated with adjuvant alone or AT-50aa died within 24–48 h. Similarly, nine out of ten mice vaccinated with AT-79aa succumbed to infection while one mouse survived the challenge. In contrast, mice vaccinated with AT-62aa showed 50% protection from lethal challenge with death occurring significantly later than in the other groups. No additional mortality was observed in this group beyond 72 hours when the mice were monitored for up to 8 days. Statistical analysis was performed by Log-Rank (Mantel-Cox) test demonstrating high significance for protection afforded by AT-62aa when compared to both AT-50aa and control mice (*P* = 0.0002) and when compared to AT-79aa (*P* = 0.0043) (Figure 4B).

#### Protection against USA 300 pneumonia

Since severe respiratory infections have been reported to be caused by USA300 [25], the efficacy of the vaccine candidate AT-62aa was further explored against pneumonia induced by USA300. Mice were vaccinated three times at two weeks interval with 10 µg of AT-62aa along with 20 µg of GLA-SE (n = 5) or GLA-SE alone (n = 10). Mice were bled on day 35 to determine serum antibody response and challenged on day 41 with 1.5×10^8^ CFU of USA300. On day 35, the vaccinated mice showed antibody titers (EC_50_) with a geometric mean of 4125 with a range of 2400 to 8980. Control mice showed no detectable antibody titers. While all control mice died within 20–48 h, four out of five vaccinated mice survived the challenge, highlighting the efficacy of AT-62aa against USA300 induced pneumonia (Figure 4C). Surviving mice lost 10–25% of body weight within the first 3 days of infection but recovered from weight loss, with 2 out of 4 mice even reaching original weights. All surviving animals were active by day 5.

### Antibodies to AT-62aa (AT62-IgG) protect against lethal bacteremia

**Figure 5:**
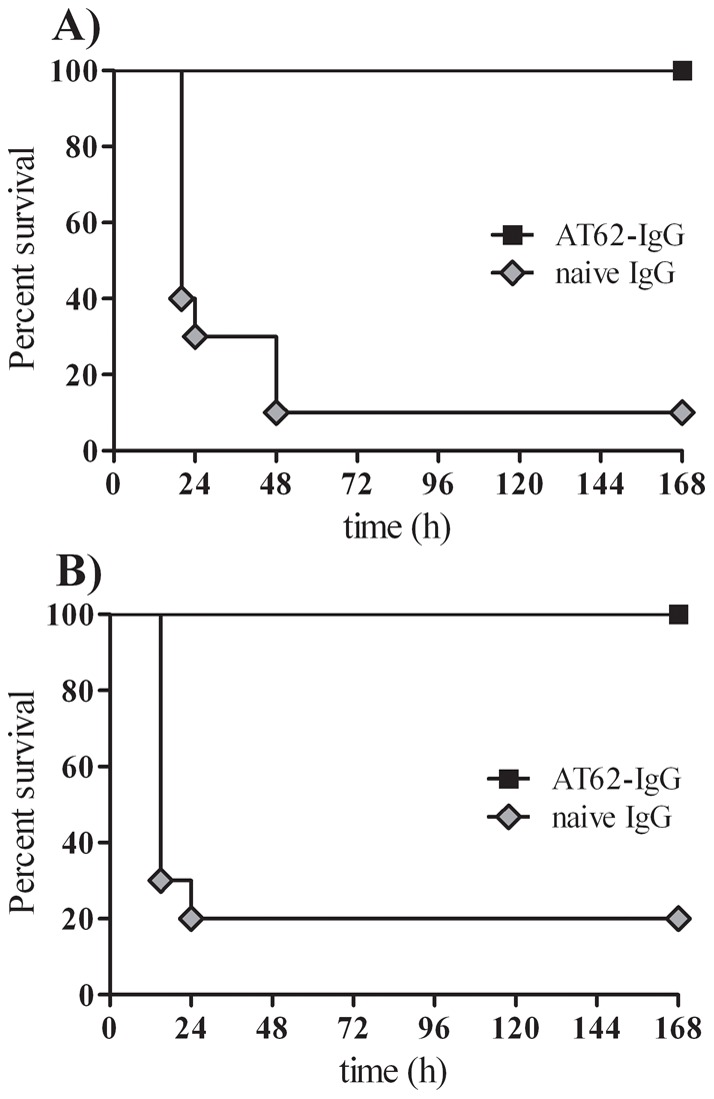
Passive protection with rabbit polyclonal AT-62aa (AT62-IgG) in bacteremia model. Protection from lethal challenge with *S. aureus* USA300 (**A**) or USA400 (**B**) after passive immunization with polyclonal rabbit antibodies AT62-IgG (black square) compared to mock-treated mice (grey diamond). Statistical analysis was performed using Log-Rank (Mantel-Cox) test, *P*<0.0001.

We hypothesized that the remarkable efficacy observed with AT-62aa vaccine is mediated by a protective antibody response. To examine this hypothesis, polyclonal antibodies were raised against purified AT-62aa in rabbits (IBT Bioservices #04–0010) and total IgG was purified by Protein A. Naïve rabbit IgG (Equitech-Bio, Inc., Kerryville, TX) was used as control. Groups of 10 mice were passively immunized with 4 mg of rabbit polyclonal anti-AT62aa antibodies (AT62-IgG) or naïve rabbit IgG by IP injection at 24 hours before challenge with 5×10^4^ CFU of USA300 or 1×10^5^ CFU of USA400 (MW2) in 3% Hog mucin. As shown in Figure 5A, while nine out of ten mice immunized with naïve IgG succumbed within 48 hours, all mice immunized with AT62-IgG showed slightly ruffled coat, remained active, and survived the challenge. Similarly, while 80% of control mice challenged with MW2 died within 48 h, all mice receiving AT62-IgG survived the lethal challenge (Figure 5B).

### Antibodies to AT-62aa protect against bacterial dissemination and organ seeding

To explore the ability of antibodies induced by the vaccine candidate AT-62aa to inhibit bacterial dissemination and/or growth *in vivo*, two studies were performed using passive immunization with AT62-IgG in pneumonia and bacteremia models.

**Figure 6:**
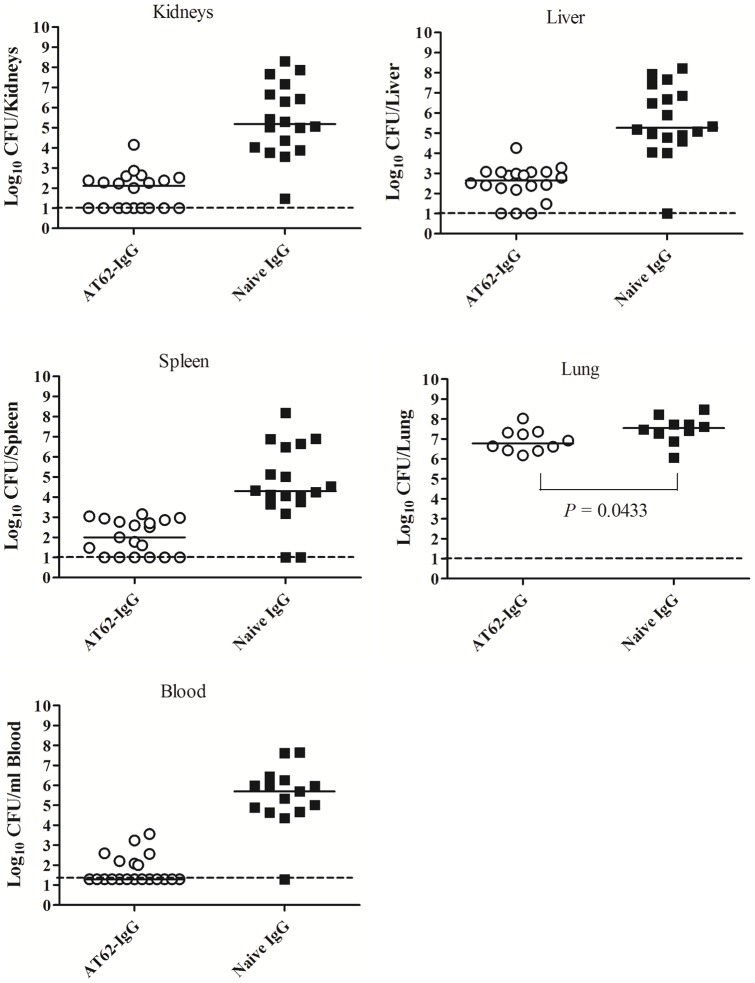
Passive protection against bacterial dissemination in bacteremia model. Bacterial burden in organs and blood was determined after passive immunization with polyclonal AT62-IgG or naïve IgG followed by IP infection with *S. aureus* USA300 in 3% Hog mucin. Statistical analysis was performed with Mann-Whitney Test with two-tailed P value (*P*<0.0001 for all tested CFU).

In the first experiment, two groups of 20 mice were passively immunized, one group with 4 mg of naïve IgG and the other group with 4 mg of AT62-IgG. After 24 hours, mice were infected IP with 5×10^4^ CFU of USA300 in 3% Hog mucin. Twelve hours after infection mice were bled, euthanized, and various organs were sampled and homogenized. In this experiment, two of the control mice died before the 12 hour time point and, thus, data could be collected only from 18 control mice. All 20 mice in the AT62-IgG treated group were alive before euthanasia for organ sampling. CFUs were determined in blood and organ homogenates. Treatment with AT62-Ig resulted in drastic reduction of bacterial burden in blood, kidney, liver, spleen, and lung, compared to control naïve rabbit IgG-treated mice (Figure 6). Blood CFU counts from three of 18 samples in the control group had to be excluded from analysis since counts were above detection limit (>300 CFU/plate) and insufficient samples were available for further dilutions. Statistical analysis using Mann Whitney test showed that the differences between control and treated groups were highly significant with *P* value <0.0001 in all cases (Figure 6). These results strongly suggest that antibodies induced by the AT-62aa antigen can be protective against dissemination of bacteria *in vivo*.

**Figure 7:**
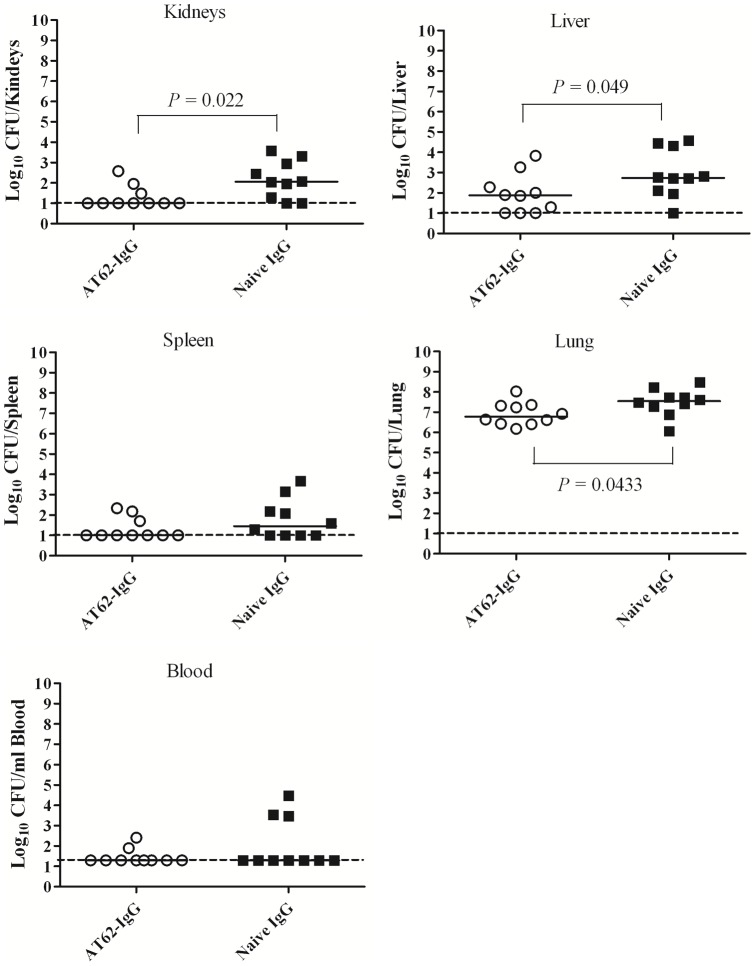
Passive protection against bacterial dissemination in pneumonia model. Bacterial burden in organs and blood was determined after passive immunization with polyclonal AT62-IgG or naïve IgG followed by IN infection with *S. aureus* USA300. Mann-Whitney Test with two-tailed P value (*P* values as indicated in figures).

In the second experiment, two groups of 10 mice were passively immunized, one group with naïve IgG and the other group with AT62-IgG (both at 4 mg/mouse). After 24 hours, mice were infected intranasally with 1.3×10^8^ CFU of USA300. Twelve hours after infection, mice were euthanized and blood and various organs were sampled and homogenized. CFUs were determined in blood and organ homogenates. Similar to results observed in bacteremia model, treatment with AT62-IgG resulted in reduction of bacterial burden in blood, kidney, liver, spleen, and lung (Figure 7). Statistical analysis using Mann Whitney test showed that the differences were significant for kidneys, liver, and lung. A trend was also observed in blood and spleen. While nine of ten control mice had infected spleens, five out of 10 mice treated with AT-62-IgG showed no bacterial seeding in spleen. In sum, antibodies induced by AT-62aa antigen can be protective against dissemination of bacteria *in vivo*.

### Mechanism of action studies

**Figure 8:**
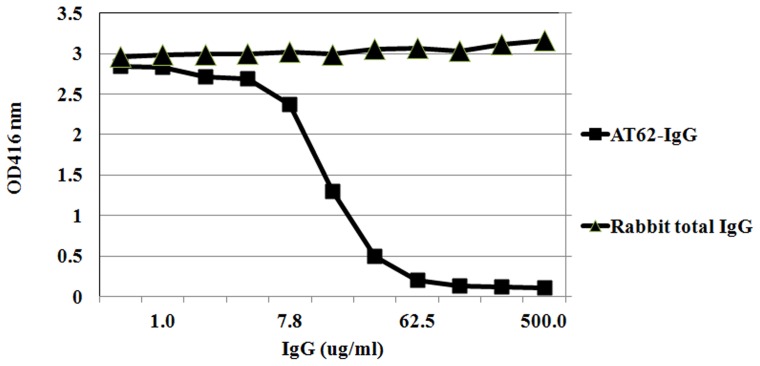
Neutralization of Hla with AT62-IgG. 100 ng of purified alpha toxin was pre-incubated at RT with different concentration of polyclonal antibody ranging from 500 µg/ml to 0.5 µg/ml and then incubated with 2% rabbit blood at 37°C for 30 min. The suspension was centrifuged and hemolysis was measured at OD_416 nm_.

To delineate the mechanism of protection by AT-62aa vaccine, we tested the effect of AT62-IgG on neutralization and oligomerization of Hla. Toxin neutralization activity of the purified polyclonal antibody was tested using rabbit red blood cells. AT62-IgG effectively inhibited RBC lysis induced by 100 ng of purified Hla (Toxin tech, FL) with an EC_50_ of 14 μg/ml (Figure 8).

**Figure 9:**
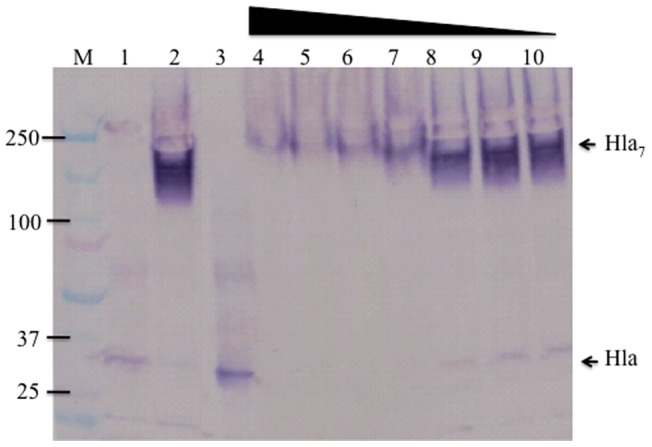
Inhibition of toxin oligomerization with AT62-IgG. Rabbit RBCs were incubated with Hla alone or Hla pre-incubated with pAb. The mixtures were incubated with 10% rabbit RBC for 45 min at 37°C, cells were pelleted, washed, lysed, and loaded in SDS-PAGE without heating. Lane 1: boiled; lane 2 at 4°C, lane 3: Hla control without RBC; lanes 4–10: 15 µg/ml of Hla neutralized with decreasing concentration of anti AT-62aa pAb (two fold diluted from 400 to 6.25 ug/ml). Western blot was developed with sheep anti-Hla polyclonal antibody.

Formation of Hla heptamers upon binding to cell surface receptor is a key event in pore formation and subsequent lysis of target cells [26]. Given the role of the N-terminal domain, we hypothesized that antibodies to AT-62aa interfere with the formation of Hla heptamers and consequently inhibit target cell lysis. To examine this hypothesis, we tested the effect of AT62-IgG on heptameric Hla (Hla7) formation in a Western blot assay. Hla was incubated with or without increasing concentrations of the pAbs before incubating the mixture with RBCs. The cells were pelleted, washed, lysed, and subjected to Western blotting without prior boiling. AT62-IgG effectively prevented the formation of the heptameric Hla structure in a dose dependent manner (Figure 9). Taken together, these data suggest that antibodies to AT-62aa neutralize the activity of Hla by binding to the N-terminal domain by preventing oligomerization and the subsequent pore formation.

## Discussion

*S. aureus* is a leading cause of community and hospital acquired infections worldwide. *S. aureus* expresses numerous virulence factors including a variety of toxins and other exoproteins that are aimed at weakening the innate immune system by direct lysis of phagocytes or inactivation of key innate response molecules, cause tissue destruction and enable bacterial dissemination leading to metastatic growth in distant organs, and provide the bacteria with much needed iron by lysis of red blood cells. Given that alpha hemolysin (Hla) plays a key role in the pathogenesis of *S. aureus* infections [8], [9], [27], this toxin represents a prime candidate for vaccine development or as a component of a multivalent *S. aureus* vaccine. However, at present, such a crucial vaccine is not available in the clinic. A great body of literature describes studies performed with an experimental Hla vaccine with a single point mutation at histidine 35. While these studies have clearly validated Hla as a prime vaccine candidate, a toxin with a single point mutation is not a viable candidate for human clinical development due to safety concerns. In this study, we used a rational, structure-based approach to design safe and effective vaccine candidates for Hla and identified a candidate with remarkable efficacy profile against *S. aureus* pneumonia and bacteremia in mouse models.

Our lead Hla vaccine candidate AT-62aa exhibits strong immunogenicity in mice when used with two clinically tested adjuvants (AlPO_4_ and GLA-SE) with significant protection shown in a mouse pneumonia model using the clinically relevant strains Newman as well as USA300 (LAC), a highly virulent CA-MRSA strain that is currently epidemic in the US. Using a mouse bacteremia model, we demonstrated the key role of Hla in the infection induced lethality, and concomitantly, showed the full protection conferred by both AT-62aa and AT-79aa against IP challenge with USA300. While an absolute cut off for protection was not established in this study, protected mice generally showed higher ELISA and neutralizing antibody titers. This finding was consistent with the ability of antibodies to AT-62aa to effectively neutralize Hla via inhibition of Hla oligomerization and suggests that toxin neutralization may be the primary mechanism of protection. This notion was supported by the ability of anti AT-62aa antibodies, passively transferred to mice before challenge, to protect against lethality and to dramatically reduce bacterial burden in different organs up to 4 logs. Hla is best known for its hemolytic activity [26] but it is also reported to induce injury to alveolar epithelial cells [28], induce the release of nitric oxide and other inflammatory mediators [29], and cause cell death in monocytes presumably through excessive IL-1β release [30]. Given this broad profile of activities of alpha toxin, AT-62aa induced neutralizing antibodies may contribute to tissue protection, contain excessive inflammatory response, protect key innate immune cells from cell death, and reduce the availability of iron for bacterial growth.

Ragle and Bubeck-Wardenburg [15] showed that monoclonal antibodies to the N-terminal domain of alpha toxin protect mice against pneumonia induced by *S. aureus* strain Newman. A purified recombinant protein comprising the first 50 amino acids of Hla protected mice against *S. aureus* pneumonia, when used for immunization with Freund's adjuvant [15]. In contrast, when we vaccinated mice with the purified protein representing this portion of Hla (AT-50aa) along with GLA-SE (Figure 3) or Alhydrogel (data not shown), very low ELISA titers were detected, neutralizing antibody titers were below 1∶40, and all AT-50aa vaccinated mice succumbed to lethal pneumonia (Figure 4B). The discrepancy with the previously reported results [15] most likely reflects the use of Freund’s adjuvant by Ragle and Bubeck-Wardenburg, which is known to be an extremely potent inducer of antibody response. However, Freund's adjuvant cannot be used in humans due to severe toxicity. When tested in bacteremia model, AT-50aa provided partial protection against lethal challenge (Figure 4A), suggesting that some level of protective response can be achieved with AT-50aa consistent with the reported results in pneumonia [15]. It must be noted that while Ragle and Bubeck-Wardenburg used a GST fusion of AT-50aa, the protein used in the current study contained only a short 6xHis tag. It is possible that the GST tag may have additional conformational impact on the protein further contributing to the observed efficacy.

The analysis of Hla crystal structure shows that the N-terminal 50 amino acids represent an incomplete domain consisting of two beta strands. Our preliminary biophysical studies using circular dichroism showed that the AT-50aa protein largely consists of alpha helices and unstructured regions with no indication of beta sheets (data not shown). The fusion construct AT-79aa showed evidence of a mixture of beta sheets and alpha helices. These structural deviations from the beta sheet structure correlate with the reduced ability of these two proteins to induce a robust protective immune response. We believe that the misfolding of AT-79aa relates to the linker region connecting the third beta sheet of the N-terminal domain to the distal F228-M234 sheet. Our initial studies suggested that increasing the length of the linker from 3 to 5 glycines improved the folding of the protein. We are currently working on further testing a variety of linkers to evaluate if a complete four beta sheet structure can be achieved and to compare its immunogenicity to AT-62aa.

A large number of pathogens exploit the tendency of the immune system to induce overwhelming antibody response to immunodominant epitopes as an immune evasion strategy [31]. This tendency, coined as “deceptive imprinting”, is hard wired in the immune system and poses a major challenge in vaccine development [32]. Previous reports and our unpublished data indicate that most people have antibody titers against Hla. In a recent study, we examined the antibody response to multiple staphylococcal toxins in 100 patients with *S. aureus* bacteremia. While all patients showed ELISA antibody titers to alpha toxin (EC_50_ range: 130–89,000) only 52% had NT_50_ titers above 40 (Range: 1–1500, Manuscript in press; Adhikari *et al*, Journal of Infectious Diseases). Importantly, in this study, an inverse correlation between neutralizing antibodies to Hla and the likelihood of sepsis could be established. It is a well established strategy in the field of vaccine design to mask the dominant epitopes and redirect the immune response towards protective subdominant epitopes [33]. The majority of antibody response to native Hla may be directed to non-neutralizing dominant epitopes. Representing a key domain in Hla olgomerization, AT-62aa may be refocusing the antibody response toward subdominant protective epitopes resulting in strong protective efficacy, a notion consistent with previous reports of protective epitopes in the N-terminal domain of alpha toxin [15]. We have generated a panel of monoclonal antibodies to AT-62aa and 30% of the clones were found to be neutralizing, suggesting a concentration of neutralizing epitopes in this domain (unpublished data). Current efforts are focused on identifying the protective epitope(s) that could be used to refine our antigen design as well as potential immunotherapy strategies for *S. aureus* infections.

Taken together, the results presented in this report demonstrate that a rationally designed subunit vaccine can yield efficacy in animal models against two major complications of *S. aureus* infections. This remarkable efficacy may relate to refocusing of the immune response to a limited number of highly protective epitopes. Together with previous reports of Hla vaccine efficacy in SSTI infection models [8], our attenuated and highly effective alpha toxin antigen must be considered as an essential component of future *S. aureus* vaccine.
